# Saraiki language characters dataset (SLCD)

**DOI:** 10.1016/j.dib.2024.110473

**Published:** 2024-05-01

**Authors:** Muhammad Ahmad Khan, Khalil Khan, Abdulrahman Aloraini, Rehan Ullah Khan

**Affiliations:** a Pak-Austria Fachhochschule: Institute of Applied Sciences and Technology, Haripur, Pakistan; b Department of Computer Science, School of Engineering and Digital Sciences, Nazarbayev University, Kazakhstan; c Department of Information Technology, College of Computer, Qassim University, Buraydah, Saudi Arabia

**Keywords:** Natural language processing, Text recognition, Machine learning, Optical character recognition

## Abstract

About 26 million people worldwide use the Saraiki language [1]. In the southern part of Punjab and Sindh, Saraiki language is extensively spoken. One of the most important Saraiki cultural hubs is Dera Ghazi Khan. In Dera Ghazi Khan, the Saraiki language is spoken by over 90 % of the population. Calligraphers use a sophisticated script to write this language. Despite the vast body of Optical Character Recognition (OCR) literature and research dedicated to other languages, a fully functional OCR system is still needed for Saraiki language [2,3]. This work presents a genuine dataset of Saraiki handwritten characters, consisting of 50,000 scanned photos, and makes it accessible to the public for use. All of the photographs include handwritten text contributed by teachers and students from Pak-Austria Fachhochschule for Applied Sciences and Technology, Pakistan. Around 1000 people, roughly half men and half women, contributed in writing this text. For scientific research, the dataset will be made accessible to the general public.

Specifications Table

**Table:** 

Subject	Computer Science, Artificial Intelligence, Computer Vision
Specific subject area	Image processing, optical character recognition, Saraiki language character recognition
Data format	Raw
Type of data	Image
Data collection	In order to maintain dialect variety, we gathered data from individuals of different regions. The pictures of characters were compiled from handwriting of institution personnel and students. Half of the thousand people who took part in the writing exercise were men and the other half were women. In order to publish and utilize the dataset for research and scientific use, all participants were provided a consent form. Five copies of each character from 1 to 10, 11 to 20, etc., were written by each participant. Each individual adds 50 characters and uses one sheet. After greyscale scanning, we converted all pictures to binary. To provide a well-rounded dataset, we did not re-assign the sheet for writing to any of the participants.
Data source location	Pak-Austria Fachhochschule: Institute of Applied Sciences and Technology, Pakistan.
Data accessibility	Repository name: Mendeley Data identification number: doi: 10.17632/tc9zv2wf2k.1 Direct URL to data: https://data.mendeley.com/datasets/tc9zv2wf2k/1

## 1 Value of the Data

• The Data is beneficial for individuals involved in computer vision and image processing, especially those focusing on text recognition systems for the Saraiki language. Professionals in both research and industry who are engaged in NLP will discover the SLCD to be valuable.
• To develop an OCR system for the Saraiki language, the provided data can be used to train machine learning models. Further research on Saraiki OCR is still required. Our ultimate aim is to create a fully operational OCR system for the Saraiki language, and this database is a crucial part of that endeavour.

## 2 Background

OCR for Saraiki language is an open research area. The main objective of this paper and the availability of this datasets is giving the users a dataset which can be used for OCR and machine translation purposes for this language. To the best of our knowledge there is no publicly available database for Saraiki language which can be used for research and this study is the first one contributing to this open research area.

## 3 Data Description

**Fig. 1:**
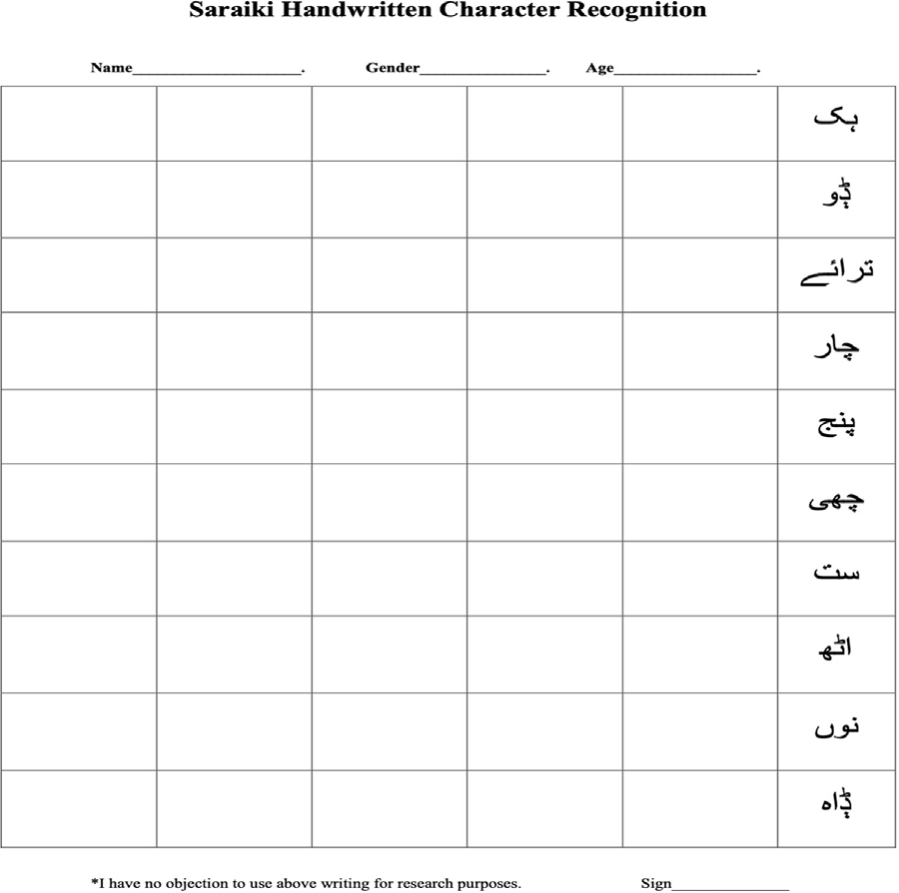
Distribution of a sample form to students, teacher and staff.

SLCD is the Saraiki handwritten characters database. Students, teachers, and staff at Pak-Austria Fachhochschule: Institute of Applied Sciences and Technology, Pakistan, were given the standardized form shown in Fig. 1. All respondents were requested to re-submit their information on paper. The text was written keeping in view the future database use; therefore it is inclusive of people of both genders. Half of the participants were male, and half were female. Once the forms were gathered, the images were scanned using an RGB colour space scanner. Data was transformed into binary form after first processing. All images in SLCD are binary images which are obtained after performing several pre-processing methods like text inclination and approach suggested in [4] involves both backward and forward scans through the label connection table, resulting in a rapid labelling process. In the preprocessing phase, the centre of each digit is identified using the connected component method, as described in [4] . Total number of images are 50,000. The images are organized into folders, each corresponding to a specific character. There are a hundred folders, each containing 500 images.

## 4 Experimental Design, Materials and Methods

Faculty and students at Pak-Austria Fachhochschule: Institute of Applied Sciences and Technology, Pakistan, were asked to write all Saraiki character on a blank piece of paper. A total of 1000 people participated in this drive. Half of the participants are male, and the other half are female. The planned database includes people between the ages of 18 to 60. We used a 600 DPI scanner to scan each page. Fig. 1 is an example of the form that was delivered to teachers, students, and staff.

**Fig. 2:**
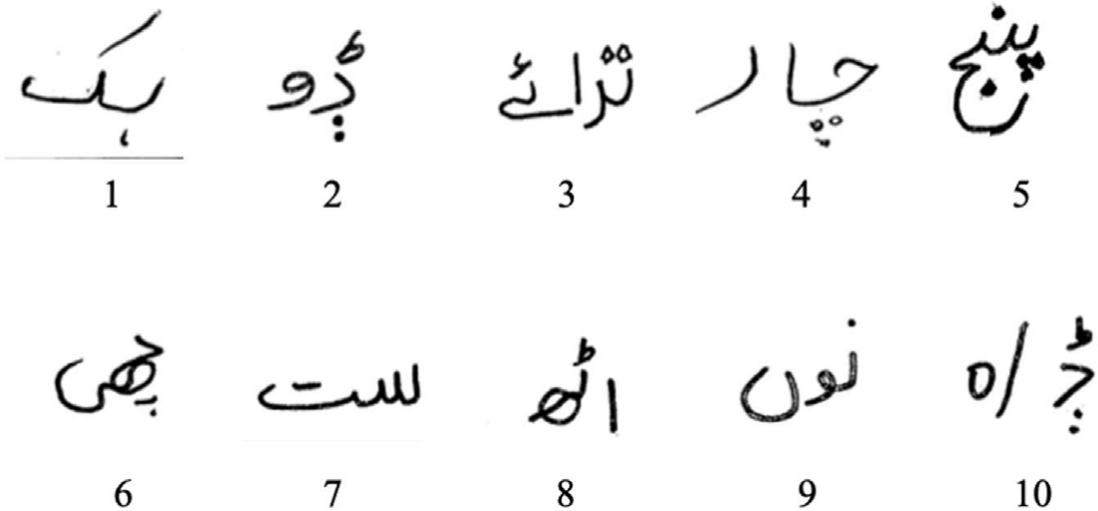
Original Text samples Collected.
• Each of the images is converted to binary format at the conclusion of the pre-processing step (see Fig. 3).

The following procedure is used to extract the characters from scanned images:

• Most existing OCR systems only support reading text in a linear format. In the case of reading creative, non-linear literature, these approaches have their limits. All these problems are resolved by adjusting the text's slant. We also used text-inclination approach [5,6]. To fix the scan's tilt, we used a horizontal histogram.
• The provisional labels in most central labelling algorithms tend to spread through the linked components in a definite manner. The approach developed in uses a one-dimensional table (also known as a connection table) to store the correspondences between the different labels used in the process. These tags spread not only to the associated parts but also to the surface of the table. In this sense, the dissemination of labels reflects the connectedness between labels (provisional or otherwise) at a given geometric distance. In most cases, this method will reduce the number of scans required. The suggested technique scans the label connection table in both directions in a sequential fashion. This makes the process of labelling items quite quick. Each character's centre was identified using the same preprocessing technique, the linked component approach.
• Each applicant writes down 10 characters each page, five times and we received the handwritten text on paper shown in Fig. 2. In the suggested pre-processing step, we performed re-scaling with size 28 ∗ 28 to extract the appropriate characters from the scanned and picture.

**Fig. 3:**
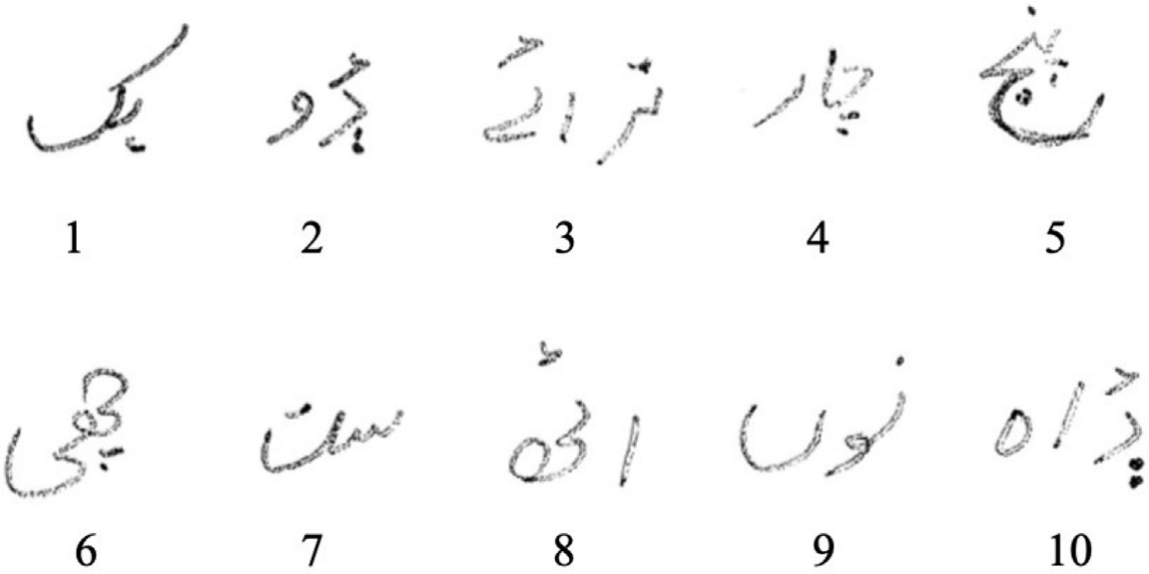
Pre-Processed SLCD.

## Limitations

Not applicable.

## Ethics Statement

All individuals who contributed to the creation of these handwritten texts provided their informed permission. Each person included in this dataset gave their permission by signing a consent form. It's also important to stress that the gathered photographs reveal no personally identifiable details about the subjects.

## CRediT authorship contribution statement

**Muhammad Ahmad Khan:** Conceptualization, Formal analysis, Data curation, Writing – original draft, Visualization. **Khalil Khan:** Conceptualization, Data curation, Writing – original draft, Visualization, Project administration. **Abdulrahman Aloraini:** Writing – original draft, Project administration. **Rehan Ullah Khan:** Writing – original draft, Writing – review & editing, Project administration.

## Data Availability

Saraiki Language Characters Dataset (SLCD) (Original data) (Mendeley Data)
